# Dandruff and Baldness

**Published:** 1897-07-31

**Authors:** Leslie Phillips

**Affiliations:** Surgeon, Birmingham Skin and Urinary Hospital


### DANDRUFF AND BALDNESS.

By Leslie Phillips, M.D., Surgeon, Birmingham Skin and Urinary Hospital.

It is conceivable that Dr. Sabouraud's belief in the identity of the micro-organisms of Alopecia areata and ordinary dandruff may be modified by further investigation, but as the matter now stands the importance of dealing with dandruff as a routine measure is emphasised by the discoveries of the Paris savant. The fact that we sometimes observe a case in which profuse dandruff co-exists with a copious growth of thick hair does not invalidate the accepted belief that the commonest factor in general loss of hair is seborrhœa, nor does it modify the prognosis of any particular case under observation.

Thinning of the hair and baldness are the ultimate results of impaired nutrition, and consequent impaired vitality of the hair, induced by dandruff, but before this stage is reached we have an obvious impairment of the natural beauty of the hair. It loses its gloss and becomes dry and coarse. An opposite condition is more rarely found in which the hair becomes unduly greasy. I have a gentleman at present under my care who presents the following striking condition: The hair is habitually bathed with natural grease, and if it and the head are washed with a solution of soft soap so as to remove this fat the re-formation is so rapid that within an hour after drying the hair a very greasy stain can be obtained by lightly drawing a piece of white blotting paper over it.

The dry form of dandruff, in which it is copious enough to fall on the clothing, is annoyingly unsightly, and it is not to be forgotten that seborrhœa of the scalp may at any time set up a seborrhœal dermatitis or eczema of the face, body, or limbs.

I have used the expression, "the importance of dealing with dandruff," instead of speaking of "curing" it, because, as far as I know, no means have yet been devised of so sterilising the scalp and hair as to result in permanent cure of the condition. It is essentially a relapsing one, and one which will certainly return if treatment is not continued. It is, however, an affection which can easily be dealt with by means of a toilet process, repeated at appropriate intervals. This is the sole secret of success. Those subject to the condition must once a week or once a fortnight, according to the severity of the affection, shampoo the scalp with a soapy embrocation, and follow this by the application of some anti-seborrhœal ointment or pomade. In diseases where it is needful to continue treatment for a long period, we may rest assured that unless the remedies prescribed not only satisfactorily answer their purpose, but are agreeable to use, our patients will not continue to use them. The remedies, however, which I habitually prescribe are well liked, and consequently their use is not neglected.

I order the head to be shampooed with spiritus saponis alkalinus Hebra (made with Eau de Cologne instead of with rectified spirit). What I consider even more satisfactory and agreeable for shampooing is Mouilla, a liquid soap, the merits of which are not sufficiently known. The manufacturers make a mouilla containing crude Russian terebine (Skip-y-da), which is very luxurious to use, and which serves the purpose of gently stimulating the scalp. The shampoo should be repeated as often as may be needful to keep visible dandruff in abeyance. In severe cases at first it may be needful to shampoo every day for a few days, but the condition rapidly improves, and the intervals can be increased. Later, men and short-cropped heads may be shampooed once a week, while women with long hair may perhaps observe a longer interval.

Immediately the hair is dried, or better, before it is quite dry, some pomade containing sulphur is to be well applied to the hair. Thorough brushing carries this down to the scalp. The pomade which I have used for many years past, and always with satisfaction, is Behrendorf's Pomade Soufrée, made by Messrs. May, Roberts, and Co., of London. Besides keeping down dandruff its use increases in a marked degree the gloss, pliability, and beauty of the hair. An application once or twice a week suffices in most cases, and often there is soon observed a diminished falling and an increased vigour of growth of the hair.

